# Leptin-Mediated Sympathoexcitation in Obese Rats: Role for Neuron–Astrocyte Crosstalk in the Arcuate Nucleus

**DOI:** 10.3389/fnins.2019.01217

**Published:** 2019-11-19

**Authors:** Xuefei Liu, Hong Zheng

**Affiliations:** Division of Basic Biomedical Sciences, Sanford School of Medicine, University of South Dakota, Vermillion, SD, United States

**Keywords:** sympathetic nerve activity, obesity, glial cells, hypothalamus, leptin

## Abstract

**Introduction:**

Accumulated evidence indicates that obesity is associated with enhanced sympathetic activation. Hypothalamic leptin-mediated signaling may contribute to the exaggerated sympathoexcitation of obesity. The goal of this study was to investigate the “neuron–astrocyte” interaction affecting leptin-mediated sympathoexcitation within the arcuate nucleus (ARCN) of the hypothalamus in obese rats.

**Methods and Results:**

Obesity was induced by high-fat diet (HFD, 42% of calories from fat) in Sprague Dawley rats. Twelve weeks of HFD produced hyperleptinemia, hyperlipidemia, and insulin resistance. In anesthetized rats, microinjections of leptin into the ARCN induced increases in heart rate (HR), renal sympathetic nerve activity (RSNA), and mean arterial pressure (MAP) in both control and HFD rats. However, microinjections of leptin in HFD rats elicited higher responses of RSNA and arterial pressure than control-fed rats. It also caused the inhibition of astrocytes within the ARCN using an astrocytic metabolic inhibitor, fluorocitrate, and reduced leptin-induced sympathetic activity and blood pressure responses. Moreover, the expression of the leptin receptor in the ARCN of HFD-fed rats was significantly increased compared to rats fed a control diet. Immunohistochemistry analysis revealed leptin receptor localization from both neurons and astrocytes of the ARCN. HFD rats exhibited increased protein expression of glial fibrillary acidic protein (GFAP) in the ARCN. We also found that the expression of astrocyte-specific glutamate transporters and excitatory amino acid transporter 1 (EAAT1) and 2 (EAAT2) were decreased within the ARCN of the HFD rats. In cultured astrocytic C6 cells, 24 h of leptin treatment increased the protein expression of GFAP and reduced the expression of EAAT1 and EAAT2.

**Conclusion:**

The results suggest that central leptin signaling occurs via neuron-astrocyte interactions in the ARCN and contributing to the exaggerated sympathoexcitation observed in obese rats. The effects may be mediated by the action of leptin on regulating astrocytic glutamate transporters within the ARCN of the hypothalamus.

## Introduction

Accumulated evidence indicates that obesity is associated with enhanced sympathetic activation (Kalil and Haynes, 2012; Head et al., 2014; Vaneckova et al., 2014; Hall et al., 2019). Chronic sympathetic activation increases cardiovascular risks, such as heart failure, hypertension, arrhythmia, atherosclerosis, and subsequent mortality in obesity (Corry and Tuck, 1999; Balasubramanian et al., 2019). The central nervous system plays a critical role in integrating peripheral afferent signals to regulate sympathetic outflow and cardiovascular function (Prior et al., 2010; Ward et al., 2011). The levels of circulating leptin have been found to be elevated in the obese condition and therefore may be related to the onset and maintenance of a hyper-sympathetic state during obesity (Bell and Rahmouni, 2016). Central leptin administration increases renal sympathetic nerve activity (RSNA), arterial pressure, and heart rate (HR) in conscious animals (Matsumura et al., 2000). The precise central mechanisms linking obesity with sympathetic overactivation are not entirely understood.

Leptin is an adipose-derived hormone that links to sympathetic activation in obesity-related hypertension (Haynes et al., 1997; Lim et al., 2013; Hall et al., 2019). Importantly, leptin can cross the blood–brain barrier to interact with a leptin receptor (OBR) in the hypothalamic nuclei. Central leptin action mainly affects feeding, thermogenesis, and sympathetic activation of the kidneys, hind limbs, and adrenal glands (Rahmouni and Haynes, 2002). Leptin can exert pressor effects through its action at the hypothalamic nuclei–the arcuate nucleus (ARCN), paraventricular nucleus, and ventromedial hypothalamus–resulting in sympathetic activation in animals (Badoer and Ryan, 1999; Rahmouni and Morgan, 2007; Habeeballah et al., 2016). The OBR is widely expressed in all these hypothalamic nuclei (Ghamari-Langroudi et al., 2011). Thus far, the central hypothalamic leptin-mediated pathways have not been fully elucidated.

The hypothalamus, including the ARCN, is a heterogeneous area comprised of different types of cells, including neurons, astrocytes, oligodendrocytes, microglia, endothelial cells, and ependymal cells (Thaler et al., 2010). The function of astrocytes in the brain extends beyond providing structural and metabolic support to the neurons. Astrocytes can also affect neuronal activity in a variety of ways, including influencing the action of glutamate and other neurotransmitters (Gordon et al., 2009). Indeed, it has been recently revealed that astrocytes serve as active mediators of various complex central mechanisms (Pan et al., 2012; Marina et al., 2016). OBR has been found in both neurons and astrocytes in the hypothalamus (Hsuchou et al., 2009b) and is responsible for leptin-induced calcium signaling in astrocytes (Hsuchou et al., 2009a). Further, it has been shown that metabolic changes can alter OBR expression and astrocytic activity in obese mice (Hsuchou et al., 2009a). Pan et al. have reported that leptin actions on astrocytic cells played an essential role in affecting metabolism and neuronal activity in obesity (Pan et al., 2012). We, therefore, proposed that defining the role of astrocytes on leptin signaling would provide an understanding of the central mechanisms involved in the sympathetic over-activation exhibited during HFD-induced obesity.

## Materials and Methods

This protocol was approved by the Institutional Animal Care and Use Committee of the University of South Dakota and was conducted in accordance with the guidelines of the National Institutes of Health Guide for the Care and Use of Laboratory Animals.

### High Fat Diet-Induced Obese Rats

Male Sprague Dawley rats (130–150 g, age 6–7 weeks) were obtained from Envigo and housed in a room with a 12-h light-dark cycle with free access to water. Rats were fed with the HFD (TD 0.88137, 42% of calories are from fat, 42% of calories from carbohydrate, 15% of calories from protein, Harlan) (*n* = 30). Rats fed a standard diet served as non-HFD controls (*n* = 30). Body weight, food consumption and blood glucose were monitored weekly. The blood glucose sample was obtained by a nick on the tail and a small drop of blood was collected to measure blood glucose by a commercial handheld glucometer (Accu-Chek, Roche). Using blood samples collected from the tail vein, levels of plasma insulin, leptin (ALPCO, Salem, NH, United States), and angiotensin II (LifeSpan BioSciences, Seattle, WA, United States) were measured by commercial ELISA kits. A total of 33 plasma samples (16 from control and 17 from HFD rats) were tested. The absorbance was measured with a microplate reader at 450 nm (PerkinElmer, Waltham, MA, United States). The plasma triglyceride level was measured by a quantification kit (BioVision, Milpitas, CA, United States). The insulin sensitivity index was calculated as 1/[log (fasting insulin) + log (fasting glucose)].

Urinary norepinephrine excretion was measured as an index of overall sympathetic nerve activity. After 12 weeks of HFD, rats were placed in metabolic cages, and 24-h urine was collected, and urine volume was measured. Urinary norepinephrine concentration was measured using an ELISA kit (LifeSpan BioSciences) and calculated as urinary norepinephrine concentration multiplied by urine volume over a 24-h period.

Acute experiments and tissue collections were performed after 12 weeks of exposure to the HFD or control diet (18-week-old rats).

### *In vivo* Electrophysiological Studies

#### General Surgery for the Recording of Renal Sympathetic Nerve Activity and Arterial Pressure

Rats were anesthetized with a cocktail of urethane (0.75–1.5 g/kg, i.p) and α-chloralose (140 mg/kg, i.p). Adequate depth of anesthesia was assessed by the absence of a corneal reflex and paw withdrawal response to a noxious pinch. The femoral vein was cannulated with PE20 tubing for administration of additional anesthesia and 0.9% saline. The femoral artery was cannulated and connected to the MacLab (ADInstruments, Colorado Springs, CO, United States) for a computer-based recording of arterial pressure and HR.

The left kidney was exposed through a retroperitoneal flank incision. A renal nerve bundle was isolated from fat and connective tissue. The nerve bundle was placed on a bipolar electrode and fixed with Wacker Silgel. The electrical signal was amplified with a Grass amplifier (gain, 10,000) with high- and low-frequency cutoffs of 1,000 and 100 Hz, respectively. The rectified output from the amplifier was displayed, using the PowerLab system to record and integrate the raw nerve discharge (full-wave rectified and integrated with a 0.5 s time constant) (Kleiber et al., 2008).

Basal nerve activity was determined at the beginning of the acute experiment. The background noise was determined by the RSNA recorded at the end of the experiment after a ganglionic blocker hexamethonium (30 mg/kg, iv) injection. The value of RSNA during the experiment was calculated by subtracting the background noise from the actual recorded value. The changes of RSNA were expressed as a percentage of the basal value of RSNA.

#### Microinjections Into the ARCN

An incision was made on the midline of the scalp. The coordinates of the ARCN were 2.3 mm posterior to the bregma, 0.5 mm lateral to the midline, and 9.6–9.9 mm ventral to the dura (Harlan et al., 2011; Kawabe et al., 2013). 30 min after the surgical procedure, a microsyringe needle (0.2 mm OD) was inserted into the ARCN for drug delivery. At the end of the experiment, blue dye (2% Chicago blue, 30 nL) was injected into the brain for histological verification.

#### Microinjection Experimental Protocols

Experiment 1: In the control and HFD groups (*n* = 8–9/group), leptin (R&D Systems, Minneapolis, MN, United States) (50, 100, and 200 ng in 50 nL yielding concentrations of 1, 2, and 4 mg/ml, respectively) (Rahmouni and Morgan, 2007; Shi et al., 2015) dissolved in the vehicle consisting of artificial cerebrospinal fluid was microinjected into the ARCN. The responses of RSNA, mean arterial pressure (MAP), and HR over the following 30 min were recorded. Microinjections into the ARCN with 50 nL of vehicle were also performed and recorded accordingly.

Experiment 2: In the separate groups of control and HFD rats (*n* = 8/group), astrocytic metabolic inhibitor fluorocitrate (20 mM, 1 nmol in 50 nL, MilliporeSigma) (Pan et al., 2011) dissolved in the artificial cerebrospinal fluid vehicle was pre-microinjected into the ARCN. A total of 30 min later, leptin (200 ng in 50 nL) was microinjected into the ARCN. The responses of RSNA, MAP, and HR over the next 30 min were recorded. Microinjections into the ARCN of the vehicle (50 nL) and, 30 min later, leptin (200 ng in 50 nL) were performed. The responses of RSNA, MAP, and HR were recorded.

#### Immunohistochemistry

Under deep anesthesia with isoflurane, rats were perfused through the left cardiac ventricle with heparinized saline followed by 4% paraformaldehyde. The brain was removed, post-fixed with paraformaldehyde, and then placed in 30% sucrose. Brain sections of the ARCN (each section 30 μm thick) were cut with a cryostat according to a stereotaxic atlas and preserved in cryoprotectant.

The floating brain sections (control and HFD, *n* = 4/group) were incubated with 10% normal donkey serum for 1 h and then incubated in the primary antibody against OBR, (anti-rabbit, 1:200∼500, Abcam, Cambridge, MA, United States) with neuronal marker NeuN, (anti-mouse, 1:500, MilliporeSigma, Burlington, MA, United States) or glial marker glial fibrillary acidic protein (GFAP) (anti-mouse, 1:500, MilliporeSigma) antibody overnight at 4°C. Negative control for dual labeling included the sections with NeuN or GFAP primary antibody only. OBR block peptide and OBR siRNA (Supplementary Figure 1) was used in the dual labeling staining to test the specificity of the OBR primary antibody. On the second day, the sections were incubated with Alexa Fluor 594 donkey anti-rabbit secondary antibody and Alexa Fluor 488 donkey anti-mouse secondary antibody (1:200, Jackson ImmunoResearch, West Grove, PA, United States) for 2 h. After washing, the sections were mounted on glass slides and cover slipped with Vectashield mounting medium (Vector Laboratories, Burlingame, CA, United States). The images of OBR with NeuN or GFAP immunofluorescence, respectively, within the ARCN (sections from −2.3 to −2.8 mm to bregma) were viewed by a Leica fluorescence microscope and captured by a digital camera (Leica, Germany). Quantification of the intensity of OBR fluorescence in the ARCN area was done using NIH ImageJ software. Six sections of the ARCN were averaged for each rat. The number of GFAP immunoreactive (GFAP^+^) cells and the number of projections from GFAP^+^ cells were measured in five randomly chosen high-power (400X magnification) fields within the ARCN using ImageJ software.

#### Micropunch of the ARCN of the Hypothalamus for Protein Measurements

In the separate groups of control (*n* = 6) and HFD (*n* = 6), under deep anesthesia with isoflurane, rats were sacrificed, and brains were removed and frozen on dry ice. Serial coronal sections (100 μm/section) of the ARCN (total 15 sections, from −2.0 to −3.5 mm to bregma) were cut with a cryostat according to a stereotaxic atlas. The sections were bilaterally punched using the Palkovits and Brownstein technique (Palkovits and Brownstein, 1983). The punches were homogenized and placed in 100 μl of radioimmunoprecipitation assay (RIPA) buffer containing 1% protease inhibitor cocktail (Promega, Madison, WI, United States), and protein samples were stored at −80°C.

#### Western Blot Measurements

The total protein concentrations were measured with a bicinchoninic acid assay kit (Pierce, Rockford, IL, United States). Samples were adjusted to contain the same total protein concentrations. 4× loading buffer was added, and samples were loaded onto sodium dodecyl sulfate polyacrylamide electrophoresis gel, subjected to electrophoresis, and transferred to a polyvinylidene difluoride membrane (MilliporeSigma). Then, the membrane was incubated with primary antibody [rabbit anti-OBR (1:300, Abcam), mouse anti-GFAP (1:500, MilliporeSigma), rabbit anti-excitatory amino acid transporter 1 (EAAT1), rabbit anti-EAAT2, rabbit anti-vesicular glutamate transporter 2 (vGLUT2) (1:200, Cell Signaling, Danvers, MA, United States), or mouse anti-β-actin (1:1000, Santa Cruz Biotechnology, Santa Cruz, CA, United States)] overnight. After the incubation with secondary antibody conjugated with fluorescent dye (1:10000, Thermo Fisher Scientific), the membrane was visualized by the Odyssey Imaging System (LI-COR Biosciences, Lincoln, NE, United States). The intensity of the band was quantified using NIH ImageJ software. The protein expression was calculated as the ratio of the intensity of the protein to the intensity of β-actin.

### *In vitro* Studies

#### GFAP Protein and Glutamate Transporter Levels in Response to Leptin in Astrocytes

Stock cultures of the astrocytic cell line C6 were purchased from American Type Culture Collection (ATCC CCL-107, Manassas, VA, United States). Cells were grown in 60 mm culture dishes in ATCC-formulated F-12K medium supplemented with 2.5% fetal bovine serum and 15% horse serum, and they were maintained at 37°C and 5% CO_2_ until 60–70% confluence before treatment with leptin. Cells were then maintained in the medium without serum for differentiation purposes. Subsequently, cells were treated with leptin at the concentration of 25–200 ng/ml or vehicle for 24 h. Each treatment was performed for four times (*n* = 4). Cultured cells prepared were subjected to immunohistochemistry study for GFAP and OBR staining, protein extraction procedure, and Western blot studies for GFAP, EAAT1, and EAAT2 protein measurements.

### Statistical Analysis

All data are presented as Means ± SE. Statistical data analysis was performed with Graph Pad Prism 7 (GraphPad Software, La Jolla, CA, United States). For the dose response studies in the electrophysiological experiments and *in vitro* experiments, log dose-response linear analysis was used first to assess the dose-dependent responses. Values of *P* < 0.05 were considered as displaying a significant linear trend. Then factorial one-way or two-way analysis of variance (ANOVA) was used, followed by the Tukey post-test or Sidak’s post-test for multiple comparisons when appropriate, to assess the difference in response between doses and the differences between experimental groups. Other measurements were compared between the groups using two tailed unpaired Student’s *t* tests. *P* < 0.05 was considered statistically significant.

## Results

### General Characteristics of Control and HFD Rats

General characteristics of control and HFD rats used in the experiments are summarized in Table 1. Twelve weeks of HFD increased animal body weight, retroperitoneal fat pad weight, and epididymal fat pad weight. The HFD rats also had increased brown adipose tissue weight. The levels of plasma leptin, insulin, and triglyceride were significantly higher in the HFD rats than in control rats. Although the plasma glucose levels were not significantly different between the groups, the HFD rats had a lower mean insulin sensitivity index. These data confirmed that 12 weeks of HFD induced hyperlipidemia, hyperleptinemia, hyperinsulinemia, and insulin resistance.

**TABLE 1:** General characteristics of control and HFD rats.

	**Control (*n* = 16)**	**HFD (*n* = 17)**		**Control (*n* = 16)**	**HFD (*n* = 17)**
Body weight (g)	415 ± 10	454 ± 17^∗^	Plasma glucose (mmol/L)	4.7 ± 0.6	5.5 ± 0.9
Retroperitoneal fat pad (g)	4.3 ± 0.5	8.7 ± 1.2^∗^	Plasma insulin (mU/L)	14.3+2.7	78.0 ± 15.6^∗^
Epididymal fat pad (g)	4.3 ± 0.2	9.0 ± 1.2^∗^	Insulin sensitivity index	0.54 ± 0.06	0.38 ± 0.04^∗^
Brown adipose tissue (g)	0.28 ± 0.03	0.61 ± 0.08^∗^	Plasma leptin (ng/ml)	358 ± 37	2577 ± 356^∗^
Plasma triglyceride (mmol/L)	1.1 ± 0.3	5.3 ± 1.3^∗^	Plasma angiotensin II (pg/ml)	92.5 ± 34.1	298.5 ± 31.4^∗^
Basal MAP (mmHg)	93 ± 4	103 ± 5	Basal heart rate (beat/min)	347 ± 26	366 ± 19
Basal Int. RSNA (μv.s)	2.2 ± 0.3	4.2 ± 0.4^∗^	24 h urinary NE (μg)	2.0 ± 0.3	6.2 ± 0.8^∗^

*Values are mean ± SE. ^∗^*P* < 0.05 vs. control group. MAP, mean arterial pressure; Int RSNA, integrated renal sympathetic nerve activity; NE, norepinephrine.*

As can be seen in Table 1, the level of plasma angiotensin II was significantly elevated, while the basal RSNA and 24-h urinary norepinephrine levels were significantly increased in the HFD group, suggesting that overall sympathetic activity was elevated. The basal MAP was also significantly increased in the HFD rats compared to control rats. There was no significant difference in HR in the two groups.

### Sympathetic Responses to Leptin Injections Into the ARCN of the HFD Rats

**FIGURE 1:**
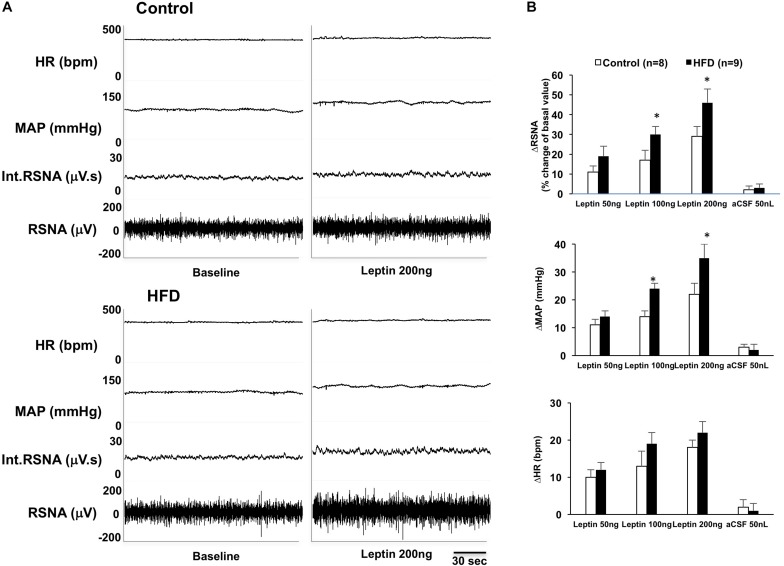
**(A)** Representative tracer of renal sympathetic nerve activity (RSNA), integrative RSNA (int.RSNA), mean arterial pressure (MAP), and heart rate (HR) responses to microinjection of leptin in the ARCN of the control and HFD rat. **(B)** Mean changes in RSNA, MAP, and HR to microinjection of leptin (50, 100, 200 ng) in the ARCN. ^∗^*P* < 0.05 vs. control group. aCSF: artificial cerebrospinal fluid.

In anesthetized rats, microinjections of leptin (50, 100, and 200 ng) into the ARCN induced dose-dependent increases of RSNA [linear regression analysis, control: *F*(1,2) = 94.83, *R*^2^ = 0.979, *P* = 0.010; HFD: *F*(1,2) = 43.05, *R*^2^ = 0.956, *P* = 0.022], MAP [control: *F*(1,2) = 31.25, *R*^2^ = 0.940, *P* = 0.031; HFD: *F*(1,2) = 48.51, *R*^2^ = 0.960, *P* = 0.020], and HR [control: *F*(1,2) = 12.27, *R*^2^ = 0.890, *P* = 0.042; HFD: *F*(1,2) = 25.95, *R*^2^ = 0.929, *P* = 0.036] in both control and HFD rats (Figure 1). Leptin administration (200 ng) in the ARCN of HFD rats elicited significantly higher increases in RSNA and MAP (reaching RSNA: 46 ± 7%, ΔMAP: 35 ± 5 mmHg) compared with control rats (ΔRSNA: 29 ± 5%, ΔMAP: 22 ± 4 mmHg) [RSNA: *F*(7,7) = 5.656, *P* = 0.036; MAP: *F*(7,7) = 2.265, *P* = 0.034]. There was no significant difference in the increase in HR seen in control and HFD groups. Vehicle injections did not affect the sympathetic responses in either group.

### Pre-inhibition of Astrocytes With Astrocytic Metabolic Inhibitor Fluorocitrate Reduced Leptin-Induced Sympathetic Responses in the ARCN

**FIGURE 2:**
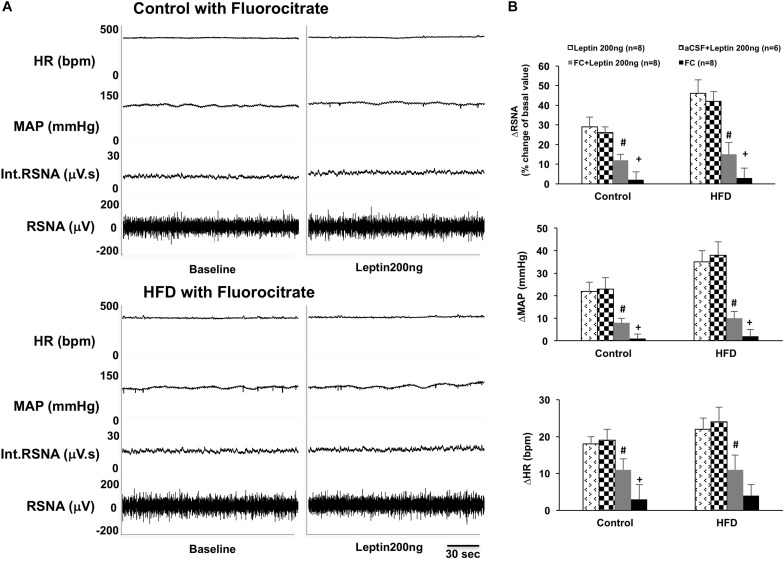
**(A)** Representative tracer of RSNA, int.RSNA, MAP, and HR responses to microinjection of leptin with fluorocitrate in the ARCN of the control and HFD rat. **(B)** Mean changes in RSNA, MAP, and HR to microinjection of leptin (200 ng) with and without fluorocitrate in the ARCN. # *P* < 0.05 vs. the leptin group without fluorocitrate. +*P* < 0.05 vs. the FC + leptin group. FC: fluorocitrate.

Inhibition of astrocytes in the ARCN with the astrocytic metabolic inhibitor fluorocitrate significantly inhibited leptin-induced increases of RSNA, MAP, and HR in the ARCN in both control and HFD rats (ΔRSNA: 12 ± 3% vs. 29 ± 5% in control, 15 ± 6% vs. 46 ± 7% in HFD; ΔMAP: 8 ± 2 mmHg vs. 22 ± 4 mmHg in control, 10 ± 3 mmHg vs. 35 ± 5 mmHg in HFD; ΔHR: 11 ± 3 bpm vs. 18 ± 2 bpm in control, 11 ± 8 bpm vs. 22 ± 3 bpm in HFD, *P* < 0.05) (Figure 2). The HFD rats had more reductions of RSNA, MAP, and HR responses to the leptin with fluorocitrate microinjections. Fluorocitrate itself did not affect RSNA, MAP, and HR responses in either group of rats (Figure 2).

### Increased Leptin Receptor (OBR) Expression in the ARCN of the HFD Rats

**FIGURE 3:**
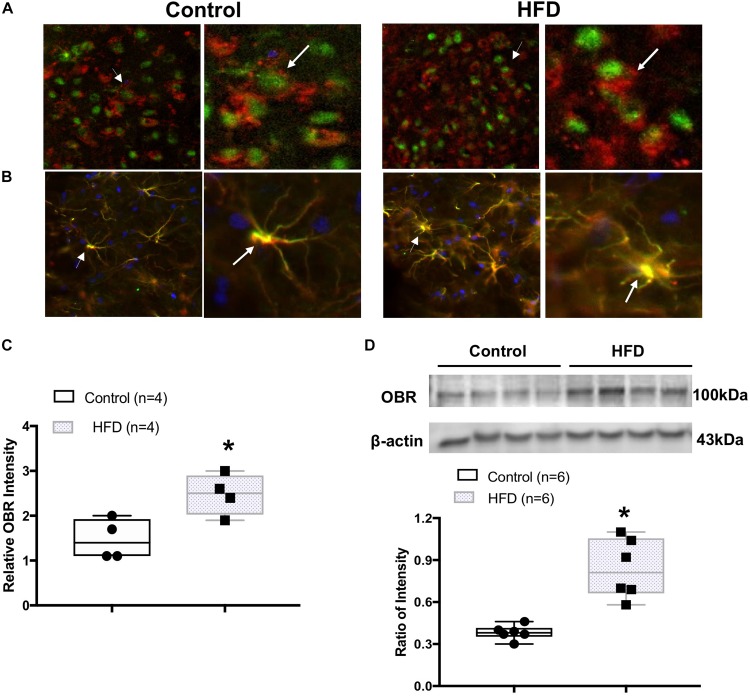
**(A)** Immunofluorescent photomicrographs from the sections of the ARCN region stained for leptin receptor (OBR, in red), neuronal marker NueN (in green), and 4′,6-diamidino-2-phenylindole (DAPI, in blue) in a control and an HFD rat (X200). Amplified images are shown in the right panel. Arrow indicates the localization of OBR in the NeuN-labeled cell. **(B)** Immunofluorescent photomicrographs from the sections of the ARCN region stained for OBR (in red), glial marker glial fibrillary acidic protein (GFAP, in green), and DAPI (in blue) in a control and an HFD rat (X200). Amplified images are shown in the right panel. Arrow indicates the colocalization of OBR and GFAP. **(C)** Mean intensity of OBR staining signal in the ARCN of control and HFD rats. **(D)** Representative gel of OBR and mean protein expressions in the ARCN of control and HFD rats. ^∗^*P* < 0.05 vs. control group.

Immunohistochemistry staining confirmed the expression of OBR within the ARCN in control and HFD rats (Figure 3). The OBR immunofluorescent signal co-localized with neuronal marker NeuN (Figure 3A) as well as with glial cell marker GFAP (Figure 3B) within the ARCN. The immunofluorescent signal for OBR was found to be increased in the ARCN from rats fed on an HFD compared to control rats (*P* = 0.021, Figure 3C). In addition, Western blot analysis showed the HFD rats had a significantly higher protein level of OBR (ratio of intensity: 0.84 ± 0.08 vs. 0.39 ± 0.02, *P* < 0.001) in the ARCN compared to the controls (Figure 3D).

### Increased Astrocyte Structural Protein (GFAP) Levels in the ARCN of the HFD Rats

**FIGURE 4:**
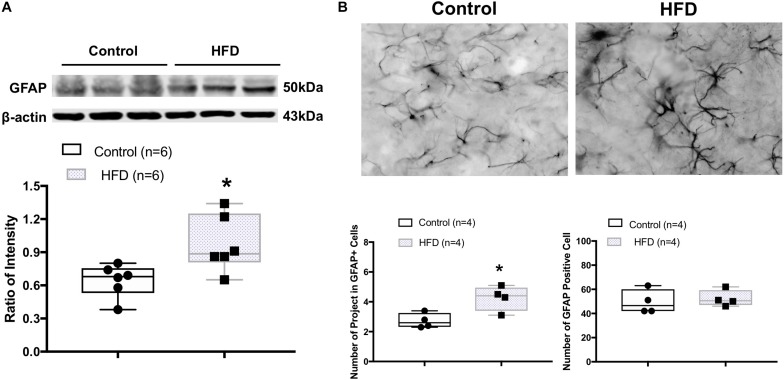
**(A)** Representative gel of GFAP and mean protein expressions in the ARCN of control and HFD rats. **(B)** Immunofluorescent photomicrographs from the sections of the ARCN region stained for GFAP positive (GFAP**^+^**) cells in a control and an HFD rat (X400). Bottom panel shows the mean number of projects in GFAP^+^ cells and mean number of GFAP^+^ cells. ^∗^**P** **<** 0.05 vs. control group.

Western blot analysis showed the HFD rats had a significantly higher protein level of GFAP (ratio of intensity: 0.97 ± 0.10 vs. 0.64 ± 0.06, *P* = 0.021) in the ARCN (Figure 4A). The morphology of GFAP^+^ cells in the ARCN of HFD rats was different from the controls (Figure 4B). Morphological analysis demonstrated that there were increases in the number of primary projections from GFAP^+^ cells in the ARCN of HFD rats (*P* = 0.021). However, there were no significant changes in the number of GFAP^+^ cells in the ARCN of HFD rats (*P* = 0.665, Figure 4B).

### Altered Glutamate Transporter Levels in the ACRN of the HFD Rats

**FIGURE 5:**
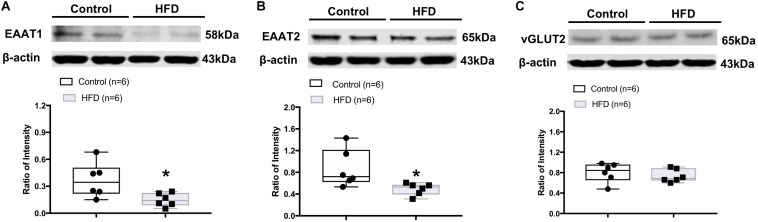
Representative gel and mean protein expressions of **(A)** excitatory amino acid transporter 1 (EAAT1), **(B)** EAAT2, and **(C)** vesicular glutamate transporter 2 (vGLUT2) in the ARCN of the control and HFD rats. ^∗^*P* < 0.05 vs. control group.

Western blot analysis showed the HFD rats had significantly lower protein levels of astrocyte-specific glutamate transporters, EAAT1 (ratio of intensity: 0.15 ± 0.03 vs. 0.34 ± 0.08, *P* = 0.026) and EAAT2 (ratio of intensity: 0.50 ± 0.06 vs. 0.86 ± 0.14, *P* = 0.031) in the ARCN (Figures 5A,B). There was no significant difference in the level of vGLUT2 protein (ratio of intensity: 0.74 ± 0.05 vs. 0.80 ± 0.08, *P* = 0.497) in the ARCN in the HFD rats (Figure 5C).

### GFAP Protein and Glutamate Transporter Levels in Response to Leptin in Astrocytes (*in vitro* Study)

**FIGURE 6:**
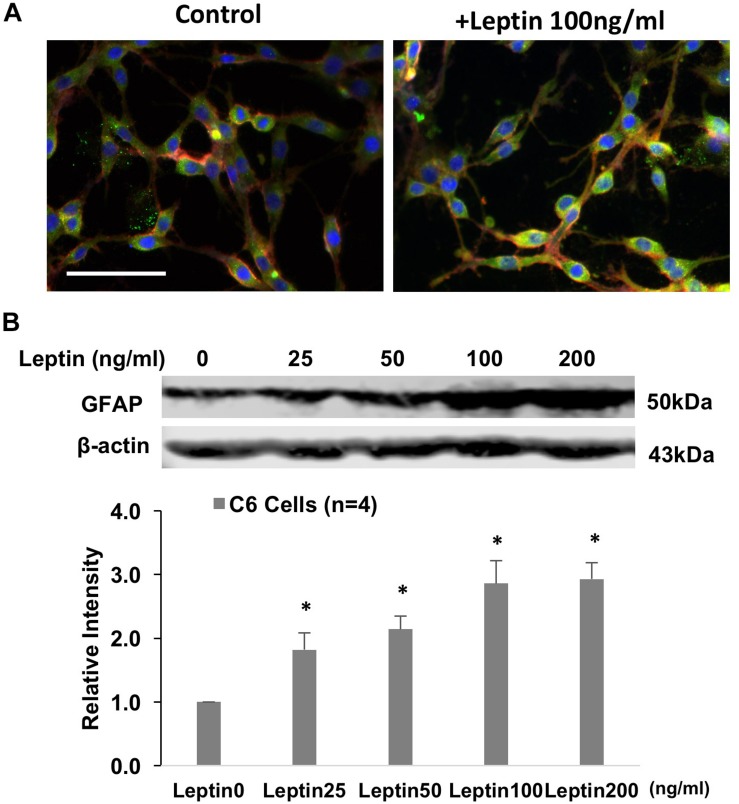
**(A)** Immunofluorescent photomicrographs from the cultured astrocytic C6 cells stained for GFAP (green), OBR (red), and DAPI (blue) with/without leptin treatment. Bar = 50 μm. **(B)**. Representative gel and mean protein expressions of GFAP in the C6 cells with leptin treatment. ^∗^*P* < 0.05 vs. control without leptin treatment.

**FIGURE 7:**
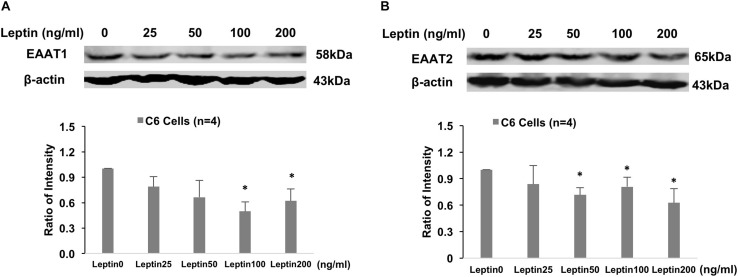
Representative gel and mean protein expressions of panels **(A)** EAAT1 and **(B)** EAAT2 in the astrocytic C6 cells with leptin treatment. ^∗^*P* < 0.05 vs. control without leptin treatment.

Immunohistochemistry staining revealed GFAP staining was increased after 24 h of leptin treatment in the cultured astrocytic C6 cells (Figure 6A). Western blot analysis showed increased GFAP protein expression with leptin incubation (25–200 ng/ml) [linear regression analysis, *F*(1,3) = 9.455, *R*^2^ = 0.759, *P* = 0.05] increasing by 2.9 folds at a concentration of 200 ng/ml [*F*(3,3) = 4.334, *P* < 0.001] (Figure 6B). After 24 h, *in vitro* exposure to leptin (25–200 ng/ml) reduced EAAT1 protein expression [linear regression analysis, *F*(1,3) = 2.442, *R*^2^ = 0.449, *P* = 0.22], decreasing it by 38% at a concentration of 200 ng/ml [*F*(3,3) = 1.34, *P* = 0.016] (Figure 7A). *In vitro* exposure to leptin also reduced EAAT2 protein expression [linear regression analysis, *F*(1,3) = 5.066, *R*^2^ = 0.628, *P* = 0.11], decreasing it by 39% at a concentration of 200 ng/ml [*F*(3,3) = 2.014, *P* = 0.022] (Figure 7B) in the astrocytes. *In vitro* exposure of leptin had no significant effects on the EAAT1 and EAAT2 protein expression in a neuronal cell line CLU (Supplementary Figure 2).

### Brain Histology

Supplementary Figure 3 depicts the brain histological verifications for the injection sites. There was a total of 38 injections within the ARCN area. Among them, 19 injection sites belonged to control group rats, and 19 injection sites belonged to the HFD rats. There were five injections that missed the ARCN area. Data from these five missed injections was excluded from the analysis.

## Discussion

In the HFD obese rat model used in this study, we observed the elevation of 24-h urinary norepinephrine excretion, which suggests an increased overall sympathetic tone in this HFD rat model. Twelve weeks of HFD also produced hyperleptinemia, hyperlipidemia, hyperinsulinemia, and insulin resistance in the rats. Hyperleptinemia, as well as higher levels of insulin and angiotensin II, have been reported to be associated with sympathoexcitation in obese conditions (Hilzendeger et al., 2012; Xue et al., 2016; Hall et al., 2019). The role of leptin in activating the sympathetic drive through the central nervous system has been highlighted in many reviews (Kalil and Haynes, 2012; Mark, 2013; Head et al., 2014). The present study confirms the critical role of leptin in relation to the sympathetic over-activation in HFD obese rats.

Leptin, an adipokine, is able to elicit a myriad of physiologic effects including, but not limited to, sympathetic activation and blood pressure regulation. The ARCN in the hypothalamus has been shown as an important gateway for the actions of leptin signaling for controlling sympathetic activity (Dampney, 2011). In our animal studies, we observed that direct leptin administration into the ARCN increased RSNA, MAP, and HR. These results were consistent with our previous study and other reports that centrally administered leptin increased sympathetic nerve activity and blood pressure (Dunbar et al., 1997; Rahmouni and Morgan, 2007; Zheng et al., 2017). More importantly, we observed that OBR protein expression in the ARCN was upregulated in the HFD rats, indicating enhanced endogenous leptin signaling after HFD feeding. Further, our electrophysiological study functionally confirmed that central stimulation with leptin resulted in elevated sympathetic activation in obese rats. Taken together, these results suggest that the up-regulation of OBR and leptin signaling within the hypothalamus could be one possible mechanism for the enhanced leptin-mediated excitatory action on sympathetic outflow elemental to the obese condition.

The hypothalamus including the ARCN is comprised of different types of cells (Gordon et al., 2009). Our dual labeling immunohistochemistry staining showed that both neurons and astrocytes in the ARCN expressed OBR. Interestingly, in the HFD obese rats, the expression of OBR was increased in the ARCN. Furthermore, we found that the HFD rats had increased levels of astrocyte structural protein GFAP and an increased number of primary projection of GFAP^+^ cells in the ARCN. This is consistent with other previous reports that increased gliosis in the ARCN is related to the enhanced leptin signaling in obesity (Pan et al., 2012). HFD-induced weight gain also results in hypothalamic gliosis and changes in the glial coverage of neurons and vasculature (Horvath et al., 2010). Our data provides further evidence of the role of astrocyte involvement in the alteration of leptin signaling. This may contribute to the leptin-mediated sympathetic over-activation in the obese condition.

Astrocytes have recently emerged as an important constituent of central sympathetic activation mechanisms (Park et al., 2009; Guo et al., 2010). It is reported the astrocytes are potential cellular substrates of angiotensin (1–7), mediating effects on local metabolism and microcirculation in the rostral ventrolateral medulla, resulting in changes in the activity of pre-sympathetic neurons and blood pressure (Guo et al., 2010). Astrocytic GABA transporters have been shown to regulate tonic GABA inhibitory function, pre-sympathetic neuronal activity, and sympathetic outflow from the paraventricular nucleus (Park et al., 2009). Indeed, in our functional study, we observed that metabolic inhibition of astrocytes significantly reduced leptin-induced RSNA, MAP, and HR. Particularly in the HFD rats, there were further reductions of RSNA, MAP, and HR after astrocytic inhibition. As described, rats in our experiments were pre-inhibited with the gliotoxin fluorocitrate, a metabolic inhibitor of reactive astrocytes. Fluorocitrate has been effectively used *in vivo* and *in vitro* to study the effects of astrocyte function on particular signaling pathways (Fonnum et al., 1997; Gordon et al., 2005). In the brain, fluorocitrate is selectively taken up by the astrocytes, blocking the activity of the enzyme aconitase, and leading to depletion of ATP stores. We did not expect fluorocitrate to produce precise changes to astrocyte remodeling. However, we anticipated that using fluorocitrate would provide a useful approach to determine the effects of altered astrocytic function in the central nervous system. In the study, the injection of fluorocitrate *per se* into the ARCN had no significant effects on sympathetic activity and blood pressure. This may support evidence that fluorocitrate does not cause significant direct effects on the excitability of neurons in the ARCN. However, further evidence needs to be provided by a direct neuronal activity recording from the ARCN.

Astrocytes participate in neuroendocrine function partially through the modulation of synaptic input density in the hypothalamus (Garcia-Caceres et al., 2011). Leptin modulates synaptic input in the hypothalamus, but whether astrocytes participate in this action is not fully known. It is reported that chronic leptin exposure increases astrocytic activation in the hypothalamus (Garcia-Caceres et al., 2011). Some studies have indicated astrocytic activity may modulate neuronal uptake and leptin signaling in obese mice (Hsuchou et al., 2009a). In the central nervous system, astrocytes participate in the control of obesity by the upregulation of their associated OBRs. Progression of obesity may be associated with a shift of the neuronal predominant expression pattern to an astrocytic preference in mice (Pan et al., 2008). In the HFD rats, we observed that astrocyte structural protein GFAP was increased and that there were morphologic changes in astrocytes in the ARCN, suggesting astrocytes may play an important role in the pathology of obesity.

Glutamate, the excitatory amino acid, plays a central role in astrocyte–neuronal interactions (Simard and Nedergaard, 2004) and contributes to sympathetic activation (Li et al., 2003, 2008). Glutamate is cleared from the neuronal synapses by astrocytes via glutamate transporters, and it is then converted into glutamine, which is released and taken up by neurons (Simard and Nedergaard, 2004). Excitatory amino acid transporters, EAAT1 and EAAT2, are the major glutamate transporters; they are expressed predominantly in astrocytic cells and are responsible for glutamate uptake (Kim et al., 2011). EAATs are responsible for regulating glutamate concentration in the synaptic cleft. When the astrocyte-mediated clearance of glutamate from the extracellular space is altered, it may cause an increased concentration of the excitatory amino acid in the extracellular space (Carney et al., 2014). Dysfunction of EAAT and accumulation of excessive extracellular glutamate has been implicated in the development of several neurodegenerative diseases (Kim et al., 2011; Zhang et al., 2016). In the present study, we observed reduced EAAT1 and EAAT2 expression in the ARCN when the rats were chronically exposed to the HFD. Additionally, our *in vitro* study showed that the expression of both EAAT1 and EAAT2 was downregulated by leptin stimulation in the cultured astrocytes. These results suggest that astrocytes play important roles in the relationship of leptin signaling within the ARCN. We propose that these important roles may be due to the effects of EAATs on astrocytic glutamate uptake. Therefore, in obese rats, reduced efficacy of EAATs in the astrocytes may result in increased sympathetic activation of leptin signaling from the ARCN. It is noted that Cano et al. (2014) reported astrocytes from HFD mice showed longer and less abundant projections accompanied by the upregulation of both glutamate transporter 1 and astrocyte glutamate transporter in the area of the hippocampus (Cano et al., 2014). The differential responses of astrocytes from the hypothalamus and outside the hypothalamus may be due to their origin in a specific brain area.

In the central nervous system, leptin has shown to exert its effects on sympathetic activity and blood pressure through a number of mediators, including glutamate (Ghamari-Langroudi et al., 2011). Both leptin and glutamate are important neuromodulators in the central nervous system. A higher glutamatergic tone also has been shown in several hyper-sympathetic disease conditions, such as chronic heart failure, hypertension, and diabetes (Luo et al., 2002; Li et al., 2003, 2008). Previously, we have reported that central leptin–glutamate signaling interactions contributed to sympathetic activation (Zheng et al., 2017). The present study provides evidence that, in the obese condition, there are alterations in the astrocytic glutamate transporter expression in the ARCN. Furthermore, the *in vitro* study indicates that leptin directly inhibits the expression of EAATs, suggesting an important role for the involvement of leptin–glutamate signaling in the obese condition.

## Conclusion

These studies provide evidence that, within the hypothalamic nuclei, leptin signaling via neuron–astrocyte interactions in the ARCN may contribute to the exaggerated sympathoexcitation observed in HFD obese rats. The effects are potentially mediated by the actions of leptin on the regulation of astrocytic glutamate transporters within the ARCN of the hypothalamus.

## Data Availability Statement

The datasets generated for this study are available on request to the corresponding author.

## Ethics Statement

The animal study was reviewed and approved by Institutional Animal Care and Use Committee of the University of South Dakota.

## Author Contributions

XL and HZ conceived and designed the experiments, performed the experiments, analyzed the data, and drafted and finalized the manuscript.

## Conflict of Interest

The authors declare that the research was conducted in the absence of any commercial or financial relationships that could be construed as a potential conflict of interest.
