# Supplementary material for: Leptin-Mediated Sympathoexcitation in Obese Rats: Role for Neuron–Astrocyte Crosstalk in the Arcuate Nucleus
Source: Front Neurosci. 2019 Nov 19;13:1217. doi: 10.3389/fnins.2019.01217 (PMC6877670; doi:10.3389/fnins.2019.01217)
Supplement: Supplementary file 1 [file Presentation_1.pptx]

## Slide 1
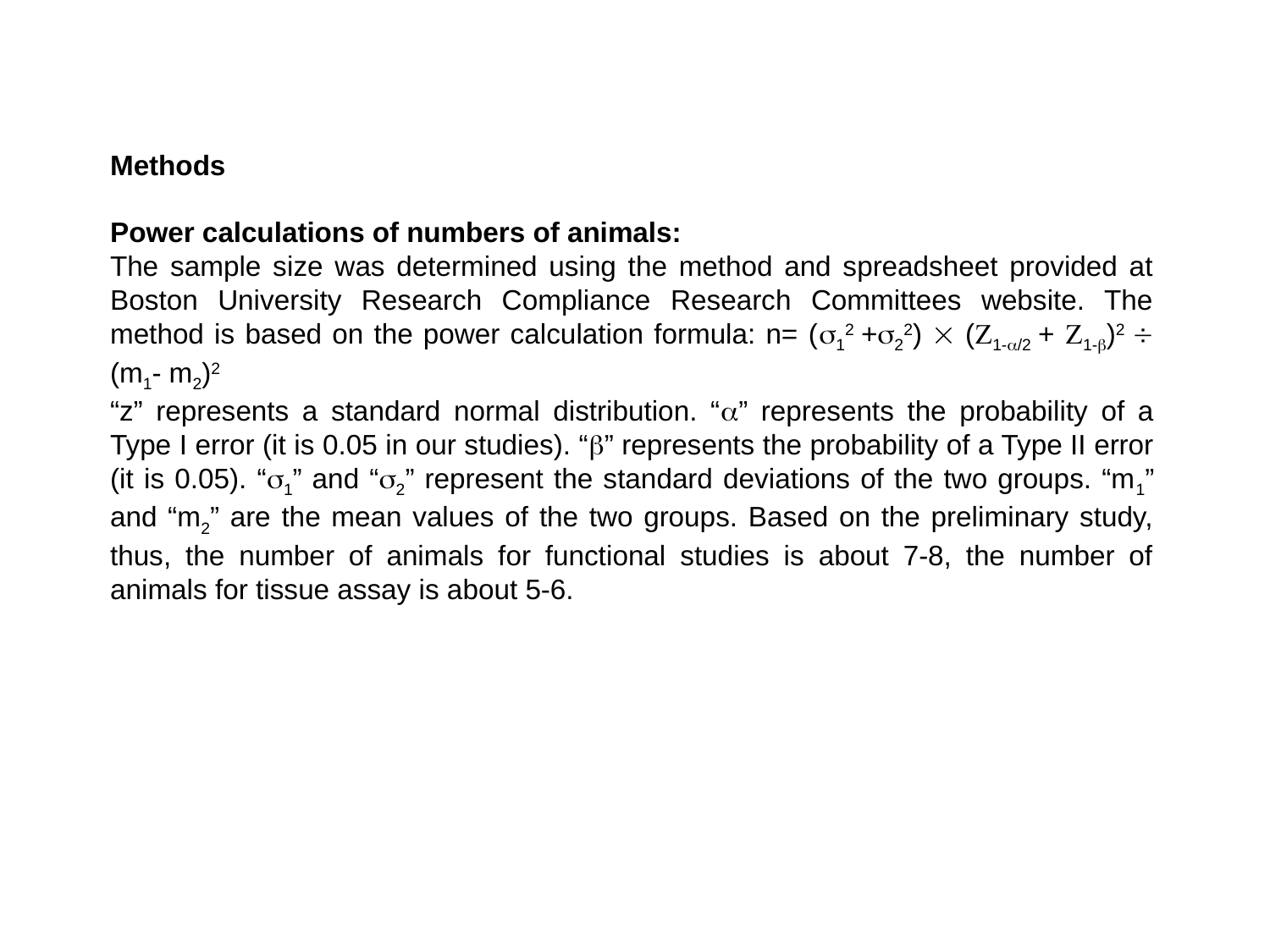

Methods
Power calculations of numbers of animals:
The sample size was determined using the method and spreadsheet provided at Boston University Research Compliance Research Committees website. The method is based on the power calculation formula: n= (σ12 +22) × (1-α/2 + 1-β)2  (m1- m2)2
“z” represents a standard normal distribution. “α” represents the probability of a Type I error (it is 0.05 in our studies). “β” represents the probability of a Type II error (it is 0.05). “1” and “2” represent the standard deviations of the two groups. “m1” and “m2” are the mean values of the two groups. Based on the preliminary study, thus, the number of animals for functional studies is about 7-8, the number of animals for tissue assay is about 5-6.

## Slide 2
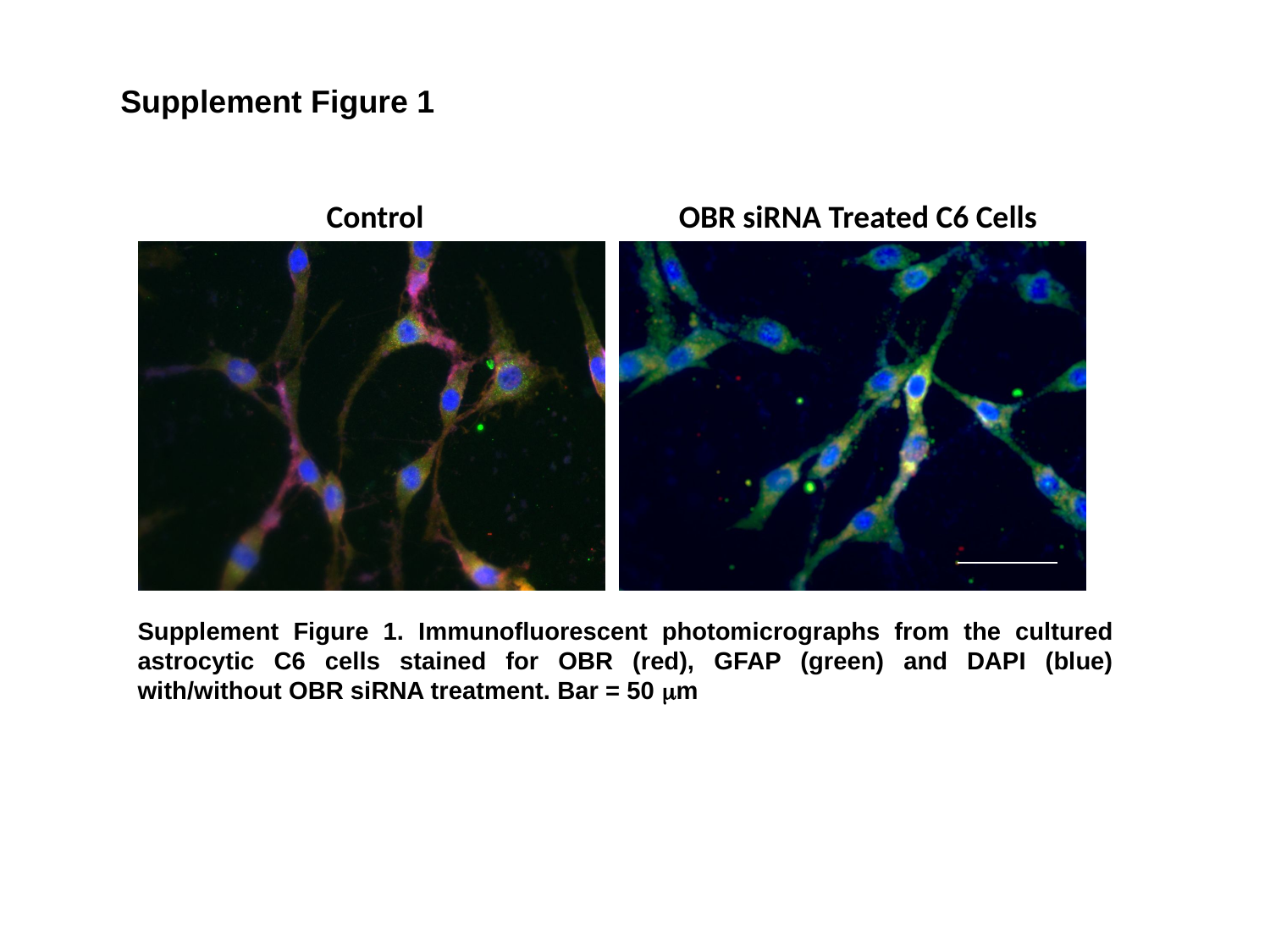

Supplement Figure 1
Control
OBR siRNA Treated C6 Cells
Supplement Figure 1. Immunofluorescent photomicrographs from the cultured astrocytic C6 cells stained for OBR (red), GFAP (green) and DAPI (blue) with/without OBR siRNA treatment. Bar = 50 μm

## Slide 3
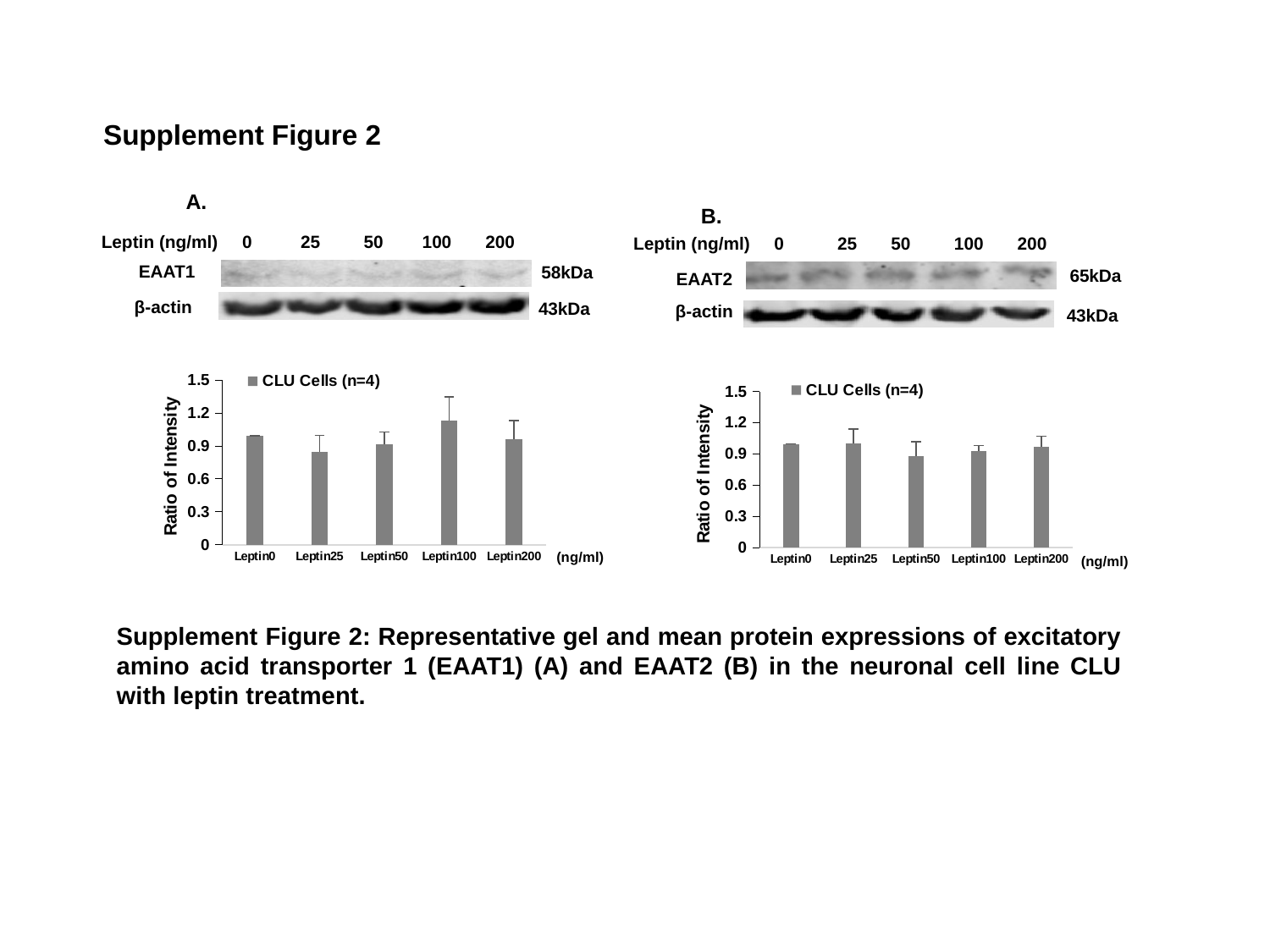

Supplement Figure 2
A.
B.
Leptin (ng/ml) 0 25 50 100 200
Leptin (ng/ml) 0 25 50 100 200
EAAT1
58kDa
65kDa
EAAT2
β-actin
43kDa
β-actin
43kDa
### Chart
| Category | |
|---|---|
| Leptin0 | 0.99084615272661 |
| Leptin25 | 0.84737286332953 |
| Leptin50 | 0.915699850180373 |
| Leptin100 | 1.128811496068892 |
| Leptin200 | 0.960015646466188 |
### Chart
| Category | |
|---|---|
| Leptin0 | 0.997603637374056 |
| Leptin25 | 1.001600047499259 |
| Leptin50 | 0.8776299882658 |
| Leptin100 | 0.932646932913828 |
| Leptin200 | 0.970854376710647 |(ng/ml)
(ng/ml)
Supplement Figure 2: Representative gel and mean protein expressions of excitatory amino acid transporter 1 (EAAT1) (A) and EAAT2 (B) in the neuronal cell line CLU with leptin treatment.

## Slide 4
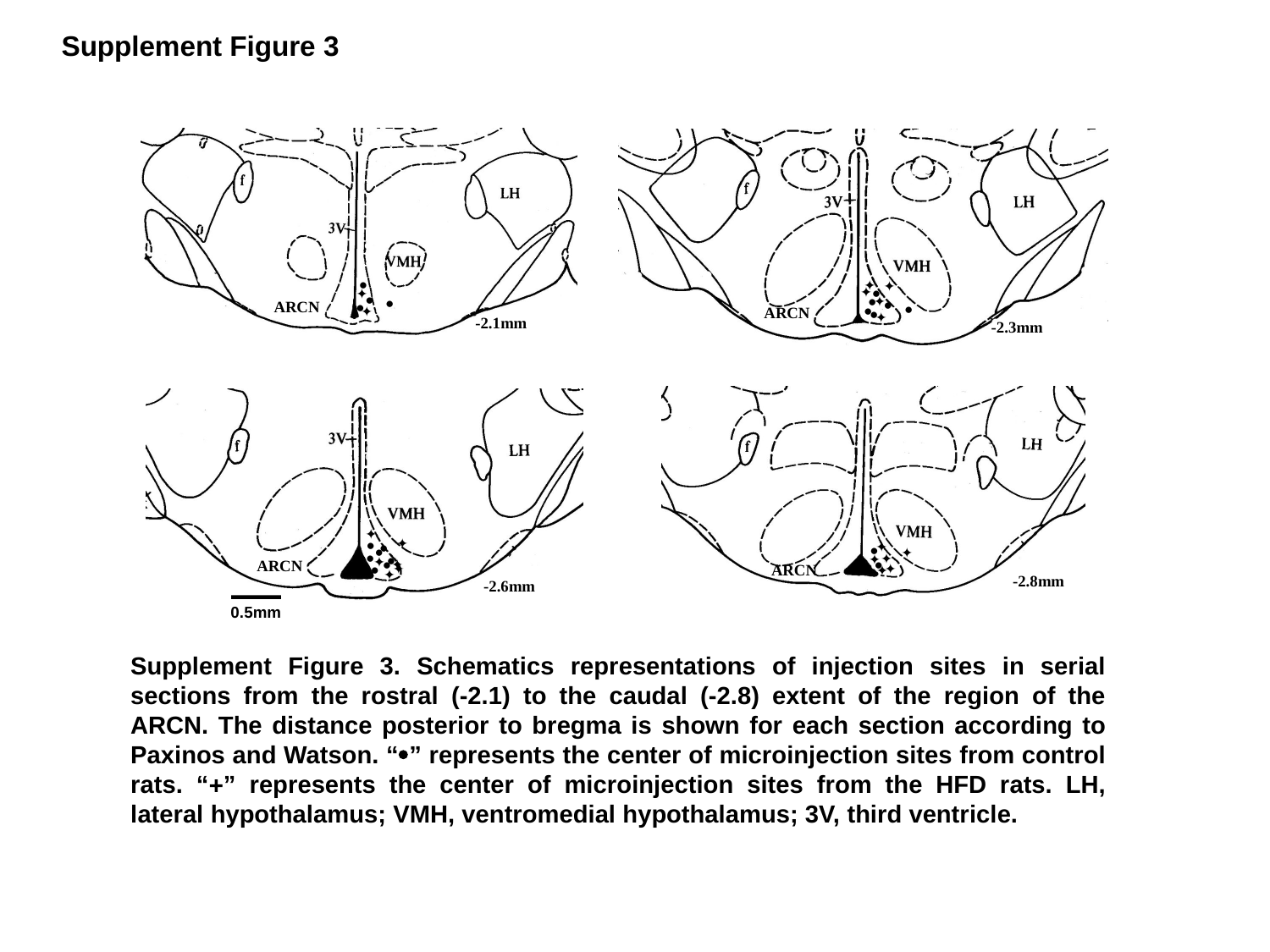

Supplement Figure 3
•
✦
✦
•
✦
✦
•
•
•
✦
•
•
ARCN
•
•
•
✦
ARCN
✦
-2.1mm
-2.3mm
✦
•
✦
•
✦
•
•
✦
•
•
✦
✦
•
✦
•
✦
ARCN
•
ARCN
✦
✦
✦
✦
-2.8mm
-2.6mm
0.5mm
Supplement Figure 3. Schematics representations of injection sites in serial sections from the rostral (-2.1) to the caudal (-2.8) extent of the region of the ARCN. The distance posterior to bregma is shown for each section according to Paxinos and Watson. “” represents the center of microinjection sites from control rats. “+” represents the center of microinjection sites from the HFD rats. LH, lateral hypothalamus; VMH, ventromedial hypothalamus; 3V, third ventricle.
